# Lymphoid and Myeloid Proliferations After Chimeric Antigen Receptor (CAR) T-Cell Therapy: The Pathologist’s Perspective

**DOI:** 10.3390/ijms26178388

**Published:** 2025-08-28

**Authors:** Jiehao Zhou, Katalin Kelemen

**Affiliations:** Department of Laboratory Medicine and Pathology, Mayo Clinic, Phoenix, AZ 85054, USA; zhou.jiehao@mayo.edu

**Keywords:** chimeric antigen receptor (CAR) T-cell therapy, lymphocytosis, immune effector cell (IEC)-associated enterocolitis, hemophagocytic lymphohistiocytosis, post-CAR T-cell lymphoma, secondary myeloid neoplasm

## Abstract

Chimeric antigen receptor (CAR) T-cell infusion has led to improved outcomes in patients with B-lymphoblastic leukemia, B-cell lymphoma, and multiple myeloma. The spectrum of post-CAR T-cell hematolymphoid abnormalities is expanding, although they remain under-recognized. Pathologists play a key role in characterizing hematolymphoid proliferation after CAR T-cell therapy. This review presents clinical and pathologic findings of common hematolymphoid proliferation after CAR T-cell therapy, illustrated by selected cases. A review of the literature is presented in the context of individual cases, and our current understanding of the pathomechanism is discussed. Infused CAR T-cells undergo a series of four phases: distribution, expansion, contraction, and persistence. In the expansion phase, transient peripheral blood lymphocytosis occurs, reaching a peak two weeks post-infusion. Delayed contraction of CAR T-cells may give rise to hemophagocytic lymphohistiocytosis-like syndrome. Immune effector cell-associated enterocolitis presents in the persistence phase, about 3–6 months after infusion. Pathologic findings include a T-cell infiltrate in the intestinal mucosa and changes resembling graft versus host disease (GVHD). This entity requires differentiation from infections and from T-cell neoplasms, including those derived from CAR T-cells. Secondary myeloid malignancies follow the same pathways as therapy-related myeloid neoplasm but present with a shorter median latency. It is essential for pathologists to recognize post-CAR T-cell hematolymphoid proliferation to support clinical decision making in a high-risk patient population.

## 1. Introduction

Chimeric antigen receptor (CAR) T-cell therapy has led to improved outcomes in patients with relapsed/refractory B-lymphoblastic leukemia, mature B-cell lymphoma, and multiple myeloma [1]. Currently, seven CAR T-cell therapies have been approved by the FDA for clinical use (Table 1). Patients receiving CAR T-cell therapy are often severely immunocompromised due to the several lines of therapy they have received. CAR T-cell infusion introduces a rapidly proliferative T-cell population with the release of various cytokines, resulting in two well-characterized, short-term outcomes of CAR T-cell therapy, namely cytokine release syndrome (CRS) and immune effector cell-associated neurotoxicity syndrome (ICANS). In addition, recipients of CAR T-cell therapy often develop lymphoid proliferations, some of which may represent transient reactive changes, while others may lead to serious adverse effects such as refractory immune effector cell-associated enterocolitis, hemophagocytic lymphohistiocytosis-like syndrome, and even secondary hematopoietic malignancies. Since CAR T-cell therapy is still in its early stages, the spectrum of recognized hematolymphoid and myeloid proliferations is expected to grow [2].

Pathologists play a key role in diagnosing lymphoid and myeloid proliferations after CAR T-cell therapy. Due to the novelty of CAR T-cell therapy, many practicing pathologists and hematologists may not have had sufficient exposure to these conditions to consistently interpret the findings and advise the clinical team. We aim to review the pathologic features and current understanding of the pathogenesis of the most common abnormal lymphoid and myeloid proliferations after CAR T-cell therapy, including atypical peripheral blood lymphocytosis, hemophagocytic lymphohistiocytosis-like syndrome, immune effector cell-associated enterocolitis, T-cell lymphoma, and secondary myeloid neoplasm. The selected case presentations illustrate these various pathological entities. Understanding the timing, clinical presentation, and pathologic findings of post-CAR T-cell abnormalities is critical in guiding clinical decision making in a high-risk patient population.

## 2. Materials and Methods

### 2.1. Cases

There were one hundred and ninety-six patients receiving CD19 and B-cell maturation antigen (BCMA) CAR-T therapy at Mayo Clinic Arizona between 1 September 2018 and 1 January 2025. Representative cases of post-CAR T-cell lymphoid and myeloid proliferations were identified in the Pathology Archives (Table 2). The diagnosis and subclassification were based on the 5th edition of the World Health Organization (WHO) classification and the 2022 International Consensus Classification (ICC). In the case where a pathologic diagnosis differed between the two classifications, both diagnoses were listed, along with the respective classification system.

### 2.2. Histologic Evaluation

Peripheral blood (PB) smears were stained with Wright stain. Bone marrow (BM) aspirate smears and biopsy touch imprint preparations were stained with Wright–Giemsa. BM biopsies were fixed in formalin and stained with hematoxylin eosin (H&E). Evaluation of morphologic dysplasia and blast count was performed on PB and BM aspirate smears. BM cellularity was evaluated on BM core biopsy specimens. Gastrointestinal and lymph node biopsies were fixed in formalin and stained with either H&E or immunohistochemical stains. Immunohistochemistry was performed using the following antibodies: ALK1 (Clone D5F3, Ventana/Roche Tissue Diagnostics, Oro Valley, AZ, USA), CD2 (clone MRQ-11, Ventana/Roche Tissue Diagnostics), CD3 (clone 2GV6, Ventana/Roche Tissue Diagnostics), CD4 (clone SP35, Ventana/Roche Tissue Diagnostics), CD5 (clone SP19, Ventana/Roche Tissue Diagnostics), CD7 (clone SP94, Ventana/Roche Tissue Diagnostics), CD8 (clones SP57 or SP239, Ventana/Roche Tissue Diagnostics), CD20 (clone L26, Ventana/Roche Tissue Diagnostics), CD30 (clone Ber-H2, Ventana/Roche Tissue Diagnostics), TCR beta F1 (clone 8A3, Fisher Scientific, Waltham, MA, USA), and TRBC1 (clone E6Z3S, Cell Signaling Technology, Danvers, MA, USA). All stains were performed on a Ventana Benchmark Ultra automatic stainer.

### 2.3. Flow Cytometric Analysis

Flow cytometric analysis of peripheral blood was conducted using antibodies specific to surface antigens commonly utilized for lymphocyte evaluation, including CD2, CD3, CD4, CD5, CD7, CD8, CD19, CD45, and TRBC1. A minimum of 50,000 total events were acquired per analysis tube on a FACSLyric flow cytometer (BD Biosciences, San Jose, CA, USA). All antibodies were procured from BD Biosciences. Analyses were performed using FACSuite RUO v1.5 software (BD Biosciences).

### 2.4. Conventional Cytogenetic and Interphase Fluorescence In Situ Hybridization (FISH) Studies

Karyotype analysis was conducted in duplicate on fresh BM aspirate samples in tissue culture medium. Chromosome preparations, including harvesting and GTW banding, followed standard methods. Cytogenetic abnormalities were classified according to the International System for Human Cytogenetic Nomenclature [3].

### 2.5. Next Generation Sequencing (NGS) for Hematologic Cancer

DNA from PB or BM aspirate samples was extracted, prepared by hybrid capture, and analyzed with NGS for tumor-associated mutations including *ASXL1*, *BRAF*, *BCOR*, *CALR*, *CBL*, *CEBPA*, *CSF3R*, *DNMT3A*, *ETV6*, *EZH2*, *FLT3*, *GATA1*, *GATA2*, *IDH1*, *IDH2*, *JAK2*, *KIT*, *KRAS*, *MPL*, *MYD88*, *NOTCH1*, *NPM1*, *NRAS*, *PHF6*, *PTPN11*, *RUNX1*, *SETBP1*, *SRSF2*, *TERT*, *TET2*, *TP53*, *U2AF1*, *WT1*, and *ZRSR2*. The panel has a sensitivity of 5–10% variant allele fraction (VAF) with a minimum depth coverage of 250× for single base substitutions and insertion/deletion events.

## 3. Cases and Discussion

### 3.1. Absolute Lymphocytosis After CAR-T-Cell Infusion

**Figure 1:**
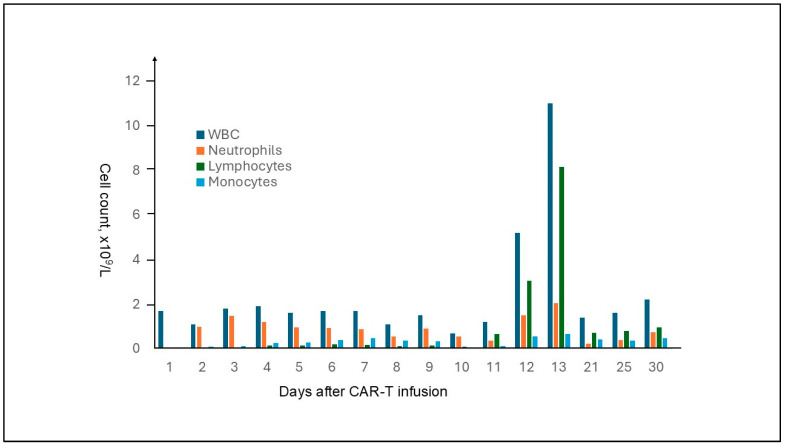
Leukocyte kinetics following BCMA CAR T-cell infusion in a patient with refractory plasma cell myeloma.

**Case report:** The patient is a 75-year-old male with refractory IgG lambda myeloma who previously received multiple lines of myeloma therapy. Before CAR T-cell infusion, he received bridging and lymphodepleting therapy. On the day of CAR T-cell infusion, the patient’s CBC showed pancytopenia. The lymphocytes were absent. His WBC remained low for the first week after CAR T-cell infusion ranging from 1.1 to 1.7 × 10^9^/L with lymphocyte count ranging from 0.02 to 0.21 × 10^9^/L. The WBC count started to rise and peaked with WBC 11 × 10^9^/L on day 13 after CAR T-cell infusion when the lymphocytes represented 74% (8.15 × 10^9^/L) of total WBC. Neutrophils and monocytes represented 18.6% (2.04 × 10^9^/L) and 6.1% (0.67 × 10^9^/L) of total WBC, respectively. The WBC count declined after day 13. On day 30, post CAR-T infusion, the WBC was 2.2 × 10^9^/L including 0.95 × 10^9^/L lymphocytes, 0.48 × 10^9^/L monocytes, and 0.73 × 10^9^/L neutrophils. Figure 1 presents the leukocyte kinetics. During the first month of CAR T-cell infusion, the hemoglobin levels ranged from 7.2 to 9.8 g/dL. The platelet levels ranged from 52 to 118 × 10^9^/L. The patient’s post-CAR T-cell complications included grade 1 CRS, pneumonitis involving the right upper lobe and right lower lobe of the lung, as well as COVID pneumonia. The patient recovered after anti-microbial treatment and supportive care. A PET-CT scan performed one month after CAR T-cell infusion showed no evidence of active myeloma. A bone marrow evaluation 5 months after CAR T-cell infusion showed no evidence of myeloma with molecular remission.

**Figure 2:**
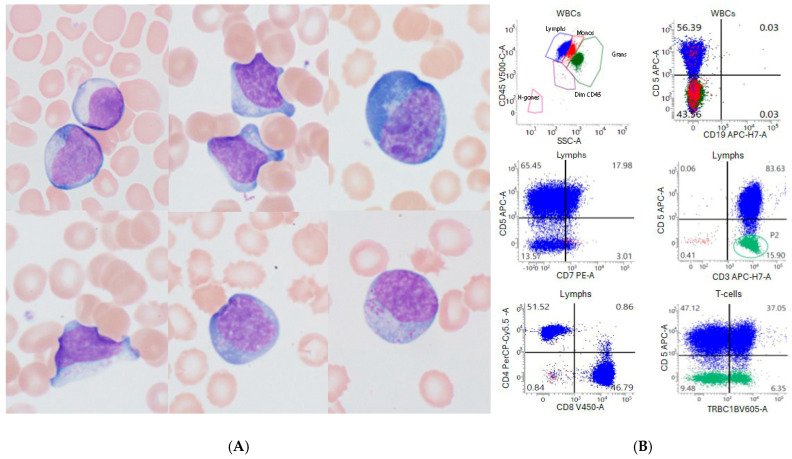
(**A**,**B**). Lymphocytosis in the peripheral blood of a patient on day 13 following BCMA CAR T-cell infusion. (**A**) Representative lymphocyte morphology (Wright stain, 1000× magnification); (**B**) immunophenotypic analysis by flow cytometry. The atypical lymphoid cells are mixed CD4+ and CD8T+ lymphocytes, CD3+/CD5+/CD7− with polytypic TRBC1 expression.

A morphology review of peripheral blood smear was performed on day 13 post-CAR-T infusion (Figure 2). The smear showed absolute lymphocytosis with atypical morphology (Figure 2A). These lymphocytes show round to irregular nucleus, occasional nucleolus, variably abundant basophilic cytoplasm, and occasional fine to coarse cytoplasmic granules. Flow cytometric analysis (Figure 2B) showed 70% lymphocytes with few granulocytes, monocytes, and basophils. The lymphocytes were composed of 98% of T-cells and rare immunophenotypically unremarkable NK cells. CD19+ B cells were absent. The T-cells were mixed CD4+ and CD8+ T-cells (CD4/CD8 ratio of 1.1:1). They expressed CD3 and CD2 with loss of CD7. A small subset of T-cells (15% of T-cells) also showed loss of CD5 expression.

**Discussion**: The lymphocytosis presented in the current case is a typical phenomenon observed in patients receiving CAR T-cell infusions. After the CAR T-cells are infused, they usually undergo a series of four dynamic phases: distribution, expansion, contraction and persistence [4,5,6,7]. After infusion, CAR T-cells circulate in the bloodstream and migrate to various organs. The distribution phase is characterized by a temporary decrease in CAR T-cells in circulation. Upon encountering malignant cells with the targeted antigen, CAR T-cells rapidly proliferate during the expansion phase, peaking typically within the second week after infusion [5,6,8]. The timing of peak absolute lymphocyte count (ALC) varies among CAR T-cell products, occurring around day 10 for anti-CD19 CAR T-cells and day 14 for anti-BCMA CAR T-cell therapy. During the proliferation phase, morphologically atypical lymphocytes are present in the peripheral blood and sometimes in the cerebrospinal fluid. The atypical morphologic features include irregular nuclear contours, prominent nucleoli, abundant basophilic cytoplasm, cytoplasmic projections, and polarized fine to coarse cytoplasmic granules [5]. Overall, the lymphocyte morphology demonstrates heterogeneity, as seen in our case. The heterogeneity is also reflected by the immunophenotype. The lymphocytes are primarily mixed CD4+ and CD8+ T-cells. Partial or complete loss of one or two T cell-associated antigens, such as CD2, CD5, and CD7, may occur. Immunophenotypic analysis, as demonstrated in our case, shows polytypic T-cell receptor beta constant 1 (TRBC1) expression in the T-cells, even in those with loss of T cell-associated antigen(s), indicating a reactive nature. A significant rise in lymphocytes suggests a strong CAR T-cell response. High absolute lymphocyte counts within two weeks of CAR T-cell infusion may lead to better outcomes in relapsed/refractory multiple myeloma patients [4].

Though lymphoid proliferation shows therapy effectiveness, it is associated with CRS, marked by a significant cytokine release. CRS can range from mild flu-like symptoms to severe, life-threatening reactions. Of note, our patient experienced grade 1 CRS during the first 2 weeks after the CAR T-cell infusion.

The CAR T-cell count typically declines during the contraction phase, which occurs 2–4 weeks after infusion. This reduction is secondary to target antigen elimination and programmed cell death. Studies have suggested that patients who respond favorably generally experience a slower rate of contraction [9]. The final phase of lymphocyte kinetics is the persistence phase, characterized by the long-term survival of memory type CAR T-cells, which can persist for months to years. Research has demonstrated a correlation between the detection of CAR T-cells at the persistent phase and lower rates of relapse, as well as longer periods of progression-free survival [10].

Briefly, CAR T-cells undergo distribution, expansion, contraction, and persistence after infusion. High peak peripheral blood lymphocytosis (>1.0 × 10^9^/L) predicts a positive anti-tumor effect. Although atypical in morphology and immunophenotype, the lymphocytes are heterogenous and reactive in nature. Conversely, uncontrolled expansion of CAR T-cells can lead to severe hyperinflammatory responses. Thus, clinical teams must closely monitor lymphocyte kinetics alongside other clinical and laboratory parameters to optimally manage the patients.

### 3.2. Immune Effector Cell (IEC)-Associated Hemophagocytic Lymphohistiocytosis-like Syndrome

**Figure 3:**
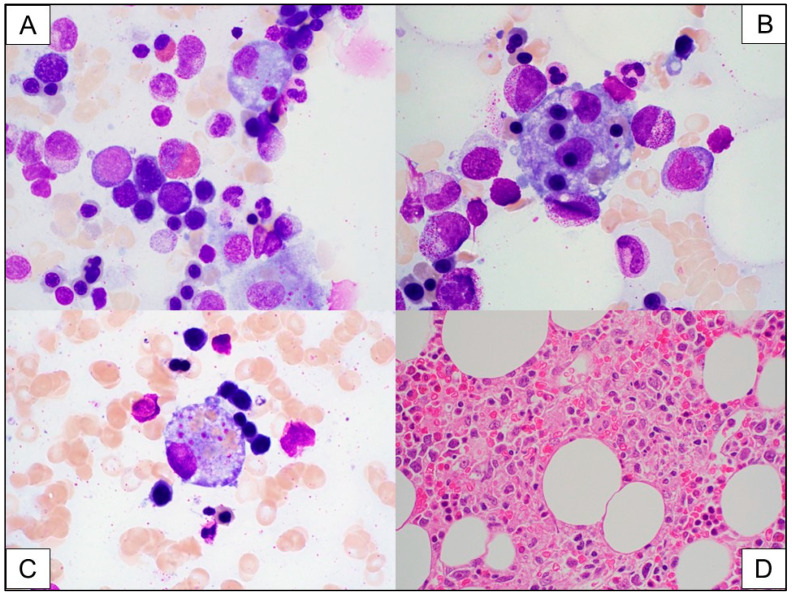
The bone marrow aspirate smears show numerous hemophagocytic histiocytes, mostly with erythrophagocytosis, and some with leuko-phagocytosis ((**A**–**C**), Wright Giemsa stain, 1000× magnification). In addition, there is prominent stress-dyserythropoiesis, with irregular nuclei and nuclear budding of the erythroid precursors (**A**,**B**). The bone marrow core biopsy shows patchy increase in histocytes, some with engulfed erythroid cells within the cytoplasm ((**D**), hematoxylin and eosin (H&E), 200× magnification).

**Case report:** A 42-year-old man with relapsed/refractory diffuse large B-cell lymphoma, T-cell histocyte-rich type, received axi-cel (Yescarta). About one month after CAR T-cell infusion, a bone marrow biopsy was performed to evaluate for persistent disease and for the etiology of prolonged pancytopenia. At the time of bone marrow biopsy, the patient had a hemoglobin of 9 g/dL, WBC of 1.6 × 10^9^/L, and platelet count of 47 × 10^9^/L. Additional abnormal blood values included an increased lactate dehydrogenase (LDH) of 2375 U/L, increased triglyceride at 693 mg/dL, and elevated ferritin that fluctuated greatly varying from 887 mcg/L to later 76,864 and finally reaching 150,000 mcg/L. Soluble interleukin 2-receptor (sIL-2R) level was markedly increased at 28,918 unit/mL (normal range 45–1105 unit/mL). The bone marrow biopsy findings are shown in Figure 3. Flow cytometric analysis demonstrated a very small persistent monotypic B-cell population, indicating low-level persistent B-cell lymphoma. The morphologic findings support the diagnosis of hemophagocytic lymphohistiocytosis (HLH). The HLH is likely secondary to CAR T-cell therapy; however, due to the low-level persistent disease, the contribution of the B-cell lymphoma to HLH cannot be ruled out.

The patient was treated with dexamethasone and etoposide based on the HLH-94 protocol [11], supported by filgrastim and transfusions. After a transient improvement, he died of multisystem failure about three months after the diagnosis of HLH.

**Discussion:** Hemophagocytic lymphohistiocytosis (HLH) is a hyperinflammatory syndrome related to uncontrolled activation of T-cells, NK-cells, and macrophages [12]. The excessive cytokine release results in complex end-organ toxicities. The common clinical and laboratory features of HLH include fever, hepatosplenomegaly, cytopenias, hypertriglyceridemia and or hypofibrinogenemia, hyperferritinemia, high soluble IL-2 receptor (soluble CD25) levels, and evidence of hemophagocytosis [13]. Recently, HLH-like clinical syndromes were described in patients who received CAR T-cell therapy [14,15,16,17,18]. Cytokine release syndrome, (CRS), another well-described hyperinflammatory syndrome after CAR T-cell therapy, shows many overlapping characteristics with HLH, since they both involve fever, cytopenias, hyperferritinemia, coagulopathy, and hypertriglyceridemia [19]. A subset of patients who experience CRS after CAR T-cell infusion may develop HLH-like features manifesting after the CRS has resolved (carHLH). This timing corresponds to the end of expansion, due to a failed or delayed contraction of CAR T-cells. Recently, the American Society of Transplantation and Cellular Therapy (ACTCT) Committee on Cellular Therapy established a working group to evaluate the underlying biology, establish diagnostic criteria, and outline treatment options of carHLH [20]. The working group proposed the terminology of Immune Effector Cell (IEC)-associated HLH.

Due to the significant overlap and the lack of specific laboratory biomarkers that could distinguish between HLH and CRS, carHLH was first considered a variant of CRS. Lately it has become clear that HLH has a different toxicity profile, has a higher need for intensive care unit admission, and may require different therapy. Several studies have reported on the timing, clinical features, and cytokine profiles of carHLH. Hines et al. reported 14.8% of carHLH in pediatric and young adult patients treated with CD19-specific CAR T-cell products [16]. Lichtenstein et al. described carHLH in a cohort of patients who received CD22-directed CAR T-cells [15]. A consistent observation across several studies was a delayed onset of carHLH at 11–14 days post-infusion (range 7–25), noticeably later than the median onset of CRS (range 3–13 days post-infusion), often when signs of CRS are resolving. For a timely laboratory diagnosis, ferritin levels are particularly useful; elevated ferritin of >10,000 ng/mL with CD19 CAR T-cells and >100,000 ng/mL with CD22 CAR T-cells, or a rapid increase in ferritin of >100 ug/L/hour within 24 hours in BCMA-targeted CAR T-cell therapy was recommended [20]. Overall, the pathophysiology of carHLH remains elusive, but it correlates with an intensified and prolonged CAR T-cell expansion and delayed CAR T-cell contraction, in concert with NK-cell lymphopenia. This suggests that a persistent NK-cell lymphopenia may contribute to an exaggerated and unchecked CAR T-cell expansion [15]. This mechanism is in line with an established role of NK-cell lymphopenia in the pathogenesis of secondary HLH [21]. Cytokine profiling studies indicate that key inflammatory cytokines are different between those patients who experienced CRS with and without carHLH. Notably, IL-1β, IFNγ, IL4, and IL-18 levels are higher in carHLH-like syndrome than in CRS [15]. This observation underlines an important role for anakinra, an IL-1 receptor antagonist in the treatment of carHLH. The therapy usually is based on initial corticosteroids, combined by either etoposide, or anakinra. Additional therapy may include ruxolitinib, a Janus kinase inhibitor, which inhibits the activity of IFNγ, IL-2, and IL-6 or other cytokine-directed therapies such as tocilizumab, an IL-6 antagonist, and emapalumab, an IFNγ antagonist.

Since the diagnosis of carHLH is based on a combination of complex abnormal laboratory findings, the role of pathology evaluation is limited in the diagnosis. Bone marrow evaluation by expert pathologists may contribute to the diagnosis by identification of hemophagocytic macrophages in the bone marrow. This finding, however, is not present consistently and is not required for diagnosis. In one study, 12 of 57 patients who had bone marrow evaluation had evidence of hemophagocytosis, 9 of which met the criteria of carHLH [15]. In another study, bone marrow evaluations were not performed at all when establishing the diagnosis of carHLH [16]. Nevertheless, pathologists have an important role in the post-CAR T-cell infusion period by monitoring bone marrow biopsies to rule out relapse of the original hematologic malignancy, or an infectious disease. Thus, pathologists may be the first to recognize hemophagocytic activity in the bone marrow.

### 3.3. Immune Effector Cell (IEC)-Associated Enteropathy

**Figure 4:**
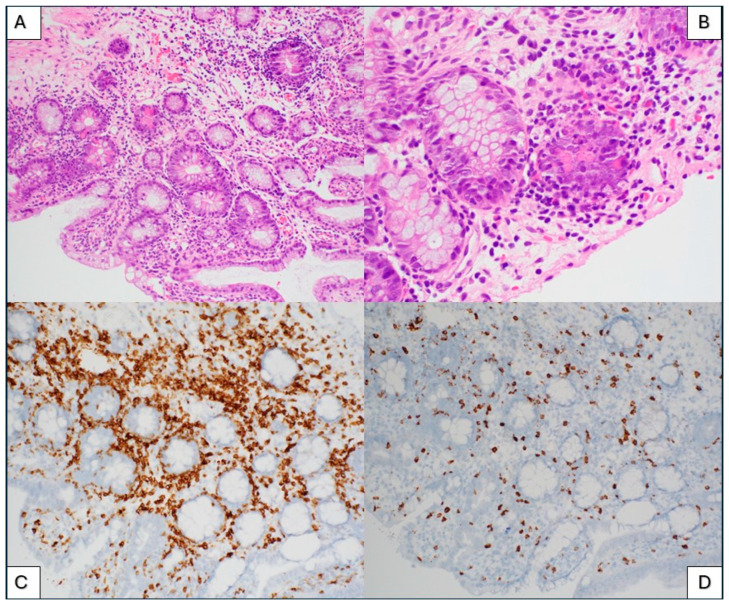
Jejunum biopsy. Hematoxylin and eosin (H&E) stained histologic sections of the jejunum biopsy show villous blunting, increased crypt epithelial apoptosis, increased eosinophils in lamina propria, lymphocytic infiltrates of the lamina propria, and increased intraepithelial lymphocytes. There is a paucity of plasma cells throughout all biopsies ((**A**), H&E, 200× magnification, (**B**), H&E 400× magnification). Immunohistochemical stains showed the majority of intramucosal lymphocytes to be CD4 positive T-cells ((**C**), 200× magnification) with CD8 positive T-cells in minority ((**D**), 200× magnification).

**Case report:** A 74-year-old woman with a history of refractory multiple myeloma underwent BCMA-directed (ide-cel) CAR T-cell therapy. Prior to lymphodepletion and CAR T-cell collection, her laboratory values included a hemoglobin of 10.2 g/uL, absolute lymphocyte count of 0.92 × 10^9^/L, and ferritin of 52 mcg/L. Five months after the CAR T-cell infusion, she developed high volume non-bloody diarrhea. Laboratory investigation for infectious microorganisms was negative for cytomegalovirus, adenovirus, Clostridium difficile, and all other agents assessed. Endoscopic evaluation showed erythematous and nodular mucosa in the gastric fundus, gastric body, and antrum, and mucosal changes in duodenum and jejunum. Histologic evaluation of the jejunum and duodenum biopsies showed villous blunting, increased crypt epithelial apoptosis, increased eosinophils in lamina propria, lymphocytic infiltrates of the lamina propria, and increased intraepithelial lymphocytes (Figure 4A,B). There was a paucity of plasma cells throughout all biopsies. The histopathologic diagnosis of immune effector cell (IEC)-induced enteritis was made.

**Discussion:** Gastrointestinal adverse effects are common after CAR T-cell therapy, affecting nearly one third of patients [22]. The main presenting feature is usually diarrhea, but abdominal pain, nausea, vomiting, fever, and abdominal distension also occur. About half of the patients have identifiable etiologies other than CAR T-cell therapy, most commonly an infection, such as C. difficile or cytomegalovirus (CMV), among others.

The largest cohort of 14 patients who developed enterocolitis after CAR T-cell therapy was reported by Fortuna et al. on a cohort of multiple myeloma patients treated with either cilta-cel (ciltacabtagene autoleucel) or ide-cel (idecabtagene vicleucel) product [23]. The overall incident was 1–2% out of more than 1200 CAR T-cell infusions. The clinical and histological presentations were similar in most cases, notably, non-bloody diarrhea beginning at least 1–3 months after the CAR T-cell infusion, corresponding to the persistence phase of CAR T-cells. Infectious microorganisms were ruled out in all cases. Histologic evaluations of gastrointestinal biopsies show a lymphocyte-predominant inflammation with a pattern resembling graft versus host disease (GVHD) after an allogeneic hematopoietic cell transplantation. The reported histologic observations usually include crypt distortion, crypt dropout, and increased apoptotic bodies. Small intestinal biopsies show villous blunting and increased intraepithelial lymphocytes. Immunohistochemical workup reveals the presence of CD3-positive T lymphocytes with absent B-cells or plasma cells. These T-cells are a mixture of CD4 and CD8 positive cells and show no significant major antigen abnormalities of CD2, CD5, or CD7. These findings are similar to the findings of our patient shown in Figure 4.

Few recent case reports have commented on clinicopathologic features of colitis after CAR T-cell therapy. Hashim et al. [24] described a female patient who presented with profuse watery diarrhea three weeks after CD19-directed CAR T-cell infusion. In this case, colon biopsies revealed a similar histologic picture of GVHD-like changes of the colon. As a contrast, another case report by Zundler et al. described a 70-year-old female who developed a profuse diarrhea of 20 loose stools per day three months after tisagenlecleucel CART-cell therapy for B-cell lymphoma [25]. In this case, the colonoscopic and histological evaluation showed a different picture, more suggestive of an inflammatory bowel disease (IBD). The authors raised the possibility of IBD-like CAR T-cell-associated colitis.

The mechanism of IEC-associated enterocolitis remains unclear. A retrospective study by Lim et al. looking for risk factors for IEC-colitis found that alkylator-based bridging therapy, a high-risk CAR-HEMATOTOX score which predicts prolonged neutropenia and high serum ferritin before and after CAR T, may be associated with increased risk [26]. Of considerable interest is the question of whether the CD3 positive T-cells that infiltrate the GI mucosa represent the CAR T-cell themselves. At least in one case, CAR-transduced T-cells were demonstrated in the lamina propria of a duodenal biopsy using antibodies to the VHH region of the cilta-cel product [23]. While this is intriguing, it does not provide evidence of the direct role of transduced CAR T-cells in the enteritis. Direct on-target toxicity cannot explain the scope of enterocolitis, though certain targets, such as BCMA, may be expressed on plasma cells and B-cells in the lamina propria of the intestine. However, BCMA expression is present in tonsils and yet, similar inflammatory infiltrates did not occur in tonsils. Chronic variable immunodeficiency (CVID)-associated colitis is also unlikely because the rapid onset of the symptoms and the lack of CVID-associated sequelae elsewhere are inconsistent with this mechanism.

Suspicious T-cell infiltrates in GI biopsies should always undergo a thorough evaluation by expert pathologists, because the boundaries between reactive lymphoid infiltrates and neoplastic lymphoid proliferations are not entirely clear. There is a single reported case of an indolent CD4+ cytotoxic T-cell lymphoma derived from CAR T-cells, which developed in the small intestine of a patient who received cilta-cel CAR T-cell therapy for multiple myeloma [27]. Immunohistochemical staining specific to CAR T-cells would be desirable in all GI biopsies suspected of IEC-associated enterocolitis. However, this may not be possible outside the setting of research. CAR T-cell constructs are unique proprietary products, and specific antibodies against the engineered T-cells are not available commercially. The partial or complete loss of certain T-cell antigens, CD2, CD5, CD7, or CD3 may be observed in T-cell neoplasms; however, they are not definitive for a diagnosis of lymphoma. TRBC1 staining and T-cell receptor gene rearrangement studies could be useful to distinguish a polyclonal CAR T-cell proliferation from emerging T-cell neoplasm. Expression of TRBC1 in either greater than 85% or less than 15% of CD3 positive T-cells can be interpreted as suggestive of T-cell clonality. Flow cytometric analysis of GI biopsies has not been used routinely due to the limited availability of tissue obtained by GI biopsy. The incidence and outcomes of CAR T-cell-derived lymphoproliferative disorders in the gastrointestinal tract are yet to be determined in the future. Pathologists have an increasing responsibility to recognize and further characterize T-cell-rich infiltrates in gastrointestinal biopsies of patients who received CAR T-cell therapy.

### 3.4. T-Cell Lymphoma After CAR T-Cell Therapy

**Case report:** A 53-year-old male with a history of chemotherapy-resistant follicular lymphoma underwent axi-cel CAR T-cell therapy. He had been in remission since then. He presented with dyspnea, diffuse lymphadenopathy, splenomegaly, and bilateral pleural effusion observed on imaging approximately 7 months later, corresponding to the persistence phase of CAR T-cell infusion. A mesenteric lymph node biopsy was performed.

**Figure 5:**
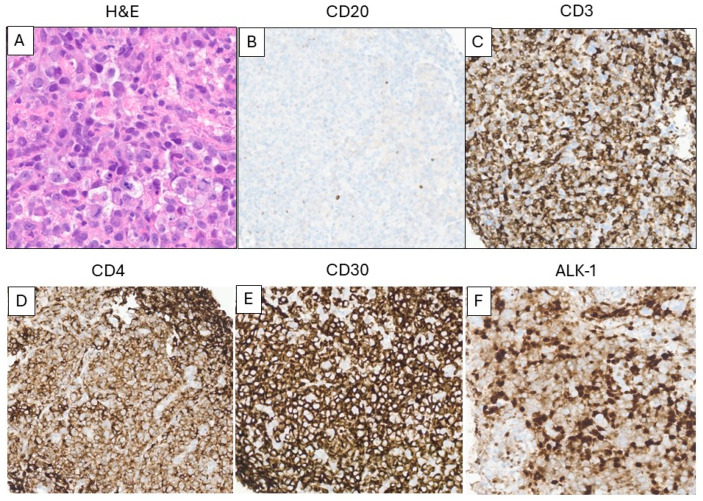
A case of ALK+ anaplastic large cell lymphoma after CAR T-cell therapy. The lymphoma cells show large size with irregular nuclei and distinct nucleoli ((**A**), H&E staining, 400× magnification). Immunohistochemical stains demonstrate the lymphoma cells are positive for CD3 (**C**), CD4 (**D**), CD30 (**E**), ALK1 (**F**), and negative for CD20 (**B**) ((**B**–**F**): immunohistochemistry, 400× magnification).

Morphological evaluation (Figure 5) showed a diffuse proliferation of atypical large lymphoid cells with round to irregular nuclei, prominent nucleoli, delicate chromatin, and variable amounts of clear cytoplasm. Increased mitosis and apoptosis were present within the atypical lymphoid cells. Immunohistochemical stains (Figure 5B–F) demonstrated a predominance of CD3 positive T-cells with an abnormal ALK-1 positive, CD30 positive, CD5 negative, CD2 partially positive, CD7 partially positive, CD4 positive, T-cell receptor alpha-beta positive, cytotoxic granule TIA1 positive, and Granzyme-B positive phenotype. The atypical T-cells showed monotypic TRBC1 expression (negative TRBC1). These combined findings were diagnostic of an anaplastic large cell lymphoma, ALK positive (ALCL). There was no diagnostic evidence of B-cell lymphoma. The patient was treated with gemcitabine/oxaliplatin, followed by etoposide and brentuximab vedotin. He developed symptoms consistent with hemophagocytic lymphohistiocytosis and died approximately three weeks after the diagnosis of ALCL.

**Discussion:** An analysis of the FDA Adverse Event Reporting System (FAERS) database identified 12,394 adverse event reports associated with CAR T-cell therapy, of which secondary primary malignancies were identified in 4.3% of the reports. T-cell malignancies were rare, representing 0.1% with 19 cases of T-cell non-Hodgkin lymphomas and two cases of large granular T-cell leukemia (T-LGL) [28]. The DESCAR-T registry, which included all pediatric and adult patients in France who had received CAR T-cell therapy since 2018, reported one instance of T-cell lymphoma (0.03%) following CAR T-cell therapy from a cohort of over 3000 patients, with a median follow-up period ranging from 12.7 to 17.7 months [29]. A review by Tix et al, combining data from 18 clinical trials and 7 studies of over 5500 patients with a median follow-up of 21.7 months, reported a secondary malignancy rate of 5.8%, with T-cell lymphoma making up only 0.09% [30]. A similar incidence of T-cell lymphoma was reported by Hamilton et al. [31]. Therefore, the incidence of T-cell lymphoma in patients receiving CAR T-cell therapy is rare, approximately 0.1%. In response to these reports, the FDA mandated that all approved CAR T-cell products include a boxed warning in their safety labeling to highlight the potential risk. The FDA also initiated investigation into the serious risk of T-cell malignancy following CAR T-cell immunotherapies.

The specific types of T-cell lymphoma identified across various studies include peripheral T-cell lymphoma, not otherwise specified (PTCL, NOS), T-LGL, and ALCL [31,32,33,34,35,36]. The lymphoma can occur as soon as a few months after CAR T-cell infusion. The diagnosis of T-cell lymphoma in patients after CAR T-cell therapy should follow the criteria provided by WHO classification, despite lacking specific guidelines.

Several risk factors have been identified for the development of post-CAR T-cell lymphoma. CAR T-cell recipients typically have a clinical history of extensive cancer treatment. Prior exposure to intensive chemotherapy, radiation therapy, and stem cell transplantation can result in DNA damage, increasing the likelihood of secondary cancers, including T-cell lymphoma. Intense inflammatory responses such as CRS and immune dysregulation associated with CAR T-cell therapy are also considered potential contributors. In individuals with previous hematopoietic malignancies, pre-existing T-cell clones with malignant potential may exist. The altered immune environment after infusion could facilitate expansion of these clones, potentially leading to secondary T-cell lymphoma. Additionally, although uncommon, some studies have detected CAR transgene integration in the lymphoma genome, suggesting that the neoplasm may be derived from engineered CAR T-cells. Since the CAR gene is delivered via retroviral vector, insertional mutagenesis related to the viral vector is considered a potential risk factor. In one case of CAR-positive T-cell lymphoma with CAR inserted in the 3′ untranslated region of *PBX2*, oncogenic mutations in *TET2*, *NFKB2*, *PTPRB*, and *JAK3* were identified [34]. Interestingly, *TET2* and *JAK3* mutations were also present in the patient’s T-cells before CAR T manufacturing. Thus, multiple risk factors may interact and contribute to T-cell lymphoma development. Unfortunately, due to limitations in the methodology for detecting CAR, it is not possible to definitively determine whether the T-cell lymphoma originated from CAR T-cells in our case.

### 3.5. Secondary Myeloid Neoplasm After CAR-T-Cell Therapy

**Figure 6:**
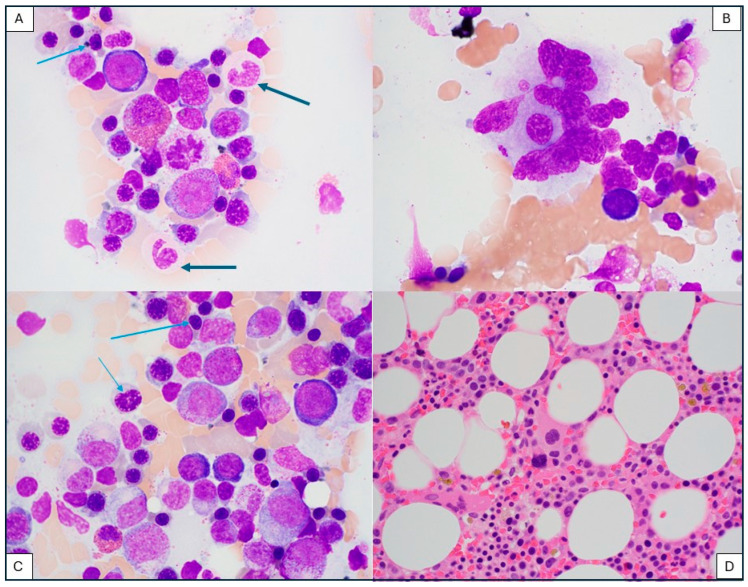
A case of post-CAR T-cell myelodysplastic syndrome. The bone marrow aspirate shows trilineage hematopoiesis with dysplastic morphologic features in neutrophils ((**A**), dark blue arrows), erythroid precursors ((**A**,**C**), pale blue arrows), and megakaryocytes (**B**). Blasts are not increased. ((**A**–**C**): Wright Giemsa, 1000× magnification). The bone marrow core biopsy shows a hypercellular bone marrow (60% cellular). A dysplastic megakaryocyte with small monolobated nucleus is shown on ((**D**), H&E, 400× magnification).

**Case report:** A 67-year-old man was diagnosed with Diffuse Large B-cell Lymphoma, Not Otherwise Specified (DLBCL, NOS), who failed several lines of combination chemotherapy and received an autologous stem cell transplantation (ASCT). When he relapsed again after ASCT, he received CD19-directed CAR T-cell therapy (axi-cel), 13 months after the original diagnosis of DLBCL. Directly after CAR T-cell treatment, he developed pancytopenia, which persisted for six months. A bone marrow biopsy was unrevealing, as it showed no evidence of lymphoma, myelodysplasia, or increased blasts. The pancytopenia persisted, and 2 years after CAR T-cell treatment a second bone marrow biopsy revealed no morphologic dysplasia but showed evidence of a single *U2AF1* mutation by next generation sequencing (NGS) with a variant allelic frequency (VAF) of 8%. The diagnosis of chronic cytopenia of undetermined significance (CCUS) was rendered and the patient was started on azacitidine 75 mg/m^2^ for 7 days every 4 weeks. After 2 years of the diagnosis of CCUS, a follow-up bone marrow biopsy showed notable dysplasia in all three hematopoietic lineages, without an increase in blasts. Karyotype analysis showed a normal male karyotype, and NGS identified *U2AF1* (VAF 8%), and a new mutation, *PMID* (VAF 24%). At this point, the diagnosis of a secondary myelodysplastic syndrome (MDS) post-cytotoxic therapy was rendered. He continued treatment with azacitidine and eltrombopag, until he suffered an accidental hip fracture. He developed complications due to heart failure and died a few weeks after the hip fracture. Figure 6 shows pathologic findings in the bone marrow aspirate at the diagnosis of myelodysplastic syndrome (Wright–Giemsa, 500×).

**Discussion:** This case shows a stepwise progression of a myeloid neoplasm progressing from CCUS to myelodysplastic syndrome (MDS) after CAR T-cell therapy. Cytopenia is common in the immediate period after CAR T-cell therapy, and it was well documented in several CAR T-cell trials [37,38,39]. The cause of immediate cytopenia includes lymphodepletion therapy and bridging chemotherapy and/or radiotherapy. Prolonged cytopenia beyond 6 months, corresponding to the persistence phase of CAR T-cells, on the other hand, requires a thorough investigation. Besides relapse of the original malignancy, infections, or emerging clonal myeloid disorders, CCUS and MDS/AML should be considered in the differential diagnosis.

Patients who undergo CAR T-cell therapy for lymphoma or multiple myeloma are heavily pretreated, raising concern for a therapy-related myeloid neoplasm. Secondary myeloid neoplasms after cytotoxic chemotherapy or radiation usually follow a predictable course with an onset time of 5–7 years after completion of alkylator therapy. The subsequent myeloid neoplasm is characterized by complex karyotype or monosomal karyotype and pathogenic *TP53* mutations [40]. Several recent case reports and case series have reported the onset of secondary MDS and AML with a much shorter onset of 3–12 months after CAR T-cell therapy [41,42,43]. A report by the Mayo Clinic based on a cohort of 189 patients who received a commercial CAR T-cell product for non-Hodgkin lymphoma, 5% developed a secondary myeloid neoplasm, with a median onset of 9.8 months [43]. Paired comparison of the cytogenetic and molecular profiles of the post-CAR-T-cell myeloid neoplasm with bone marrow findings prior to T-cell harvest documented the presence of pathogenic *DNMT3A* mutations in two patients and a *TP53* mutation in one patient in pre-CAR T-cell bone marrows. None of the *DNMT3* mutations persisted into the secondary MDS. Instead, one of them developed a *RUNX1* gene mutation and the second developed a 13q deletion. This was in contrast with a pre-existing *TP53* mutation which persisted into the post-CAR T-cell MDS phase and furthermore, the clone expanded from VAF 40% to 84%. Accorsi Buttini et al. reported post-CAR-T-cell MDS in a single case [42]. The comparative analysis of pre- and post-CAR T-cell bone marrows showed a dominant clone with *CSFR3* mutation and a subclone with *CEBPA* mutation before harvest, and the subsequent acquisition of chromosome 7 deletion and a *RUNX1* mutation at the time of the diagnosis of MDS. These observations suggest a role of cytogenetic and molecular evolution promoting the development of MDS. Falini et al. also reported molecular evolution in a single case of post-CAR-T-cell AML with complex karyotype and monosomy 7 [41]. In this case, a *PPM1D* mutation was present before the treatment, and later an additional *RUNX1* mutation occurred, possibly contributing to AML. Not all reports document molecular evolution. Vainstein et al. reported five CAR T cell-treated multiple myeloma patients who developed MDS after CAR T-cell therapy [44]. BM samples obtained prior to the CAR T-cell therapy demonstrated various MDS-related mutations, but none of the five patients developed new mutations or new cytogenetic abnormalities at the time of MDS.

While additional studies are necessary to investigate the pathomechanism of emerging clonal myeloid disorders after CAR T-cell treatment, a few important conclusions emerge. These secondary myeloid neoplasms develop with a shorter onset of 3–12 months. Certain cytogenetic and molecular abnormalities, for example, *TP53* mutation, represent a higher than usual risk for the development of clonal myeloid disorders. Therefore, it is important to perform a bone marrow evaluation with cytogenetic and molecular mutation analysis prior to T-cell harvest. Furthermore, it is imperative to discuss the additional risks with patients and to consider the bone marrow-based abnormalities when calculating overall risks and benefits of CAR T-cell therapy.

## 4. Conclusions

CAR T-cell therapy has demonstrated remarkable success in treating B lymphoblastic leukemia, multiple myeloma, and certain mature B-cell lymphomas. However, evidence of atypical hematolymphoid proliferation associated with CAR T-cell therapy is emerging. One of the most common short-term changes following CAR T-cell therapy is transient lymphocytosis, presenting as reactive atypical T lymphocytes in the peripheral blood. This lymphocytosis typically peaks within two weeks after CAR T infusion corresponding to the expansion phase of CAR T-cells, before returning to the baseline levels. While a high peak lymphocytosis is associated with positive treatment effects, Immune Effector Cell (IEC)-Associated Hemophagocytic Lymphohistiocytosis (HLH) may occur if CAR T-cell expansion is out of control or contraction is delayed. IEC-Associated HLH usually occurs 11–14 days after infusion and presents with pancytopenia and dysregulated inflammatory responses. In a subset of CAR T-cell recipients, typically 1–3 months after CAR T-cell infusion, IEC-Associated Enteropathy may develop, associated with increased intraepithelial T lymphocytes infiltrating in the gastrointestinal epithelium. Although the underlying mechanism remains elusive, limited evidence suggests it may be directly associated with infiltrating CAR T-cells in the persistence phase. Long-term adverse effects of CAR T-cell therapy include the development of secondary hematopoietic malignancies, such as T-cell lymphoma and myeloid neoplasms. Proposed pathogenesis includes insertional mutations, pre-infusion oncogenic mutations related to prior treatment, and altered immune environments. With the increased application of CAR T-cell therapies, the number of atypical hematolymphoid proliferation after CAR T-cell infusion is anticipated to increase. It is essential for pathologists to recognize these entities and to communicate with the clinical team to facilitate the most appropriate management. We hope this article provides a comprehensive review of the common abnormalities in patients receiving CAR T-cell treatment. Additionally, elucidating the underlying pathogenesis is paramount to optimizing CAR T therapies in the future.

## Figures and Tables

**Table 1:** FDA-approved CAR T-cell therapies.

CAR T-Cell Therapy	Target	Approved Use
Abecma (ide-cel)	BCMA	Multiple myeloma
Carvytki (cilta-cel)	BCMA	Multiple myeloma
Aucatzyl (obe-cel)	CD19	B-cell ALL (adult)
Kymriah (tisa-cel)	CD19	B-cell ALL (pediatric/young adult) Diffuse large B-cell lymphoma Follicular lymphoma
Tecartus (brexu-cel)	CD19	B-cell ALL (adult) Mantle cell lymphoma
Yescarta (axi-cel)	CD19	Large B-cell lymphoma Follicular lymphoma
Breyanzi (liso-cel)	CD19	Follicular lymphoma Large B-cell lymphoma Mantle cell lymphoma Chronic lymphocytic leukemia

**Table 2:** Summary of five cases of post-CAR-T abnormal lymphoid or myeloid proliferations.

Case#	Original Disease	CAR-T Type	Post-CAR-T Abnormality	Timing After CAR-T Infusion	CAR-T-Cell Phase
1	Multiple Myeloma	Celta-cel	Atypical lymphocytosis	12–14 days	Expansion
2	DLBCL	Axi-cel	IEC-associated HLH	14–30 days	Expansion/Contraction
3	Multiple Myeloma	Ide-cel	IEC-associated enterocolitis	5 months	Persistence
4	Follicular Lymphoma	Axi-cel	ALCL, ALK+	7 months	Persistence
5	DLBCL	Axi-cel	MDS	24 months	Persistence

## Data Availability

The original contributions presented in this study are included in the article. Further inquiries can be directed to the corresponding author.
